# PINK1 Interacts with VCP/p97 and Activates PKA to Promote NSFL1C/p47 Phosphorylation and Dendritic Arborization in Neurons

**DOI:** 10.1523/ENEURO.0466-18.2018

**Published:** 2018-01-10

**Authors:** Kent Z. Q. Wang, Erin Steer, P. Anthony Otero, Nicholas W. Bateman, Mary Hongying Cheng, Ana Ligia Scott, Christine Wu, Ivet Bahar, Yu-Tzu Shih, Yi-Ping Hsueh, Charleen T. Chu

**Affiliations:** 1 Department of Pathology, University of Pittsburgh School of Medicine, Pittsburgh, PA 15213; 2 Department of Cell Biology, University of Pittsburgh School of Medicine, Pittsburgh, PA 15213; 3 Department of Computational and Systems Biology, University of Pittsburgh School of Medicine, Pittsburgh, PA 15213; 4 Academia Sinica, Institute of Molecular Biology, Taipei, Taiwan 11529; 5 Pittsburgh Institute for Neurodegenerative Diseases, McGowan Institute for Regenerative Medicine, Center for Protein Conformational Diseases and Center for Neuroscience at the University of Pittsburgh, Pittsburgh, PA 15213

**Keywords:** dendritic morphology, kinase signaling, neurodegeneration, PINK1, valosin-containing protein

## Abstract

While PTEN-induced kinase 1 (PINK1) is well characterized for its role in mitochondrial homeostasis, much less is known concerning its ability to prevent synaptodendritic degeneration. Using unbiased proteomic methods, we identified valosin-containing protein (VCP) as a major PINK1-interacting protein. RNAi studies demonstrate that both VCP and its cofactor NSFL1C/p47 are necessary for the ability of PINK1 to increase dendritic complexity. Moreover, PINK1 regulates phosphorylation of p47, but not the VCP co-factor UFD1. Although neither VCP nor p47 interact directly with PKA, we found that PINK1 binds and phosphorylates the catalytic subunit of PKA at T197 [PKA_cat_(pT197)], a site known to activate the PKA holoenzyme. PKA in turn phosphorylates p47 at a novel site (S176) to regulate dendritic complexity. Given that PINK1 physically interacts with both the PKA holoenzyme and the VCP-p47 complex to promote dendritic arborization, we propose that PINK1 scaffolds a novel PINK1-VCP-PKA-p47 signaling pathway to orchestrate dendritogenesis in neurons. These findings highlight an important mechanism by which proteins genetically implicated in Parkinson’s disease (PD; PINK1) and frontotemporal dementia (FTD; VCP) interact to support the health and maintenance of neuronal arbors.

## Visual Abstract

**Figure:**
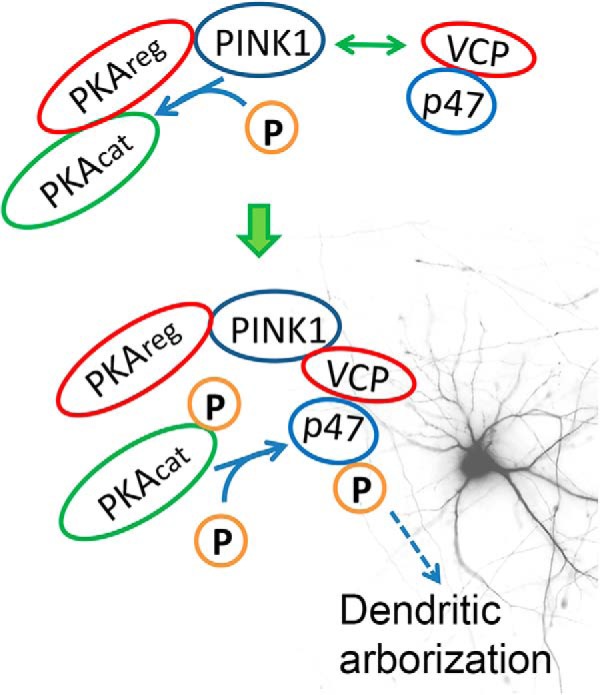

## Significance Statement

This study delineates a novel molecular mechanism by which PTEN-induced kinase 1 (PINK1) and valosin-containing protein (VCP) interact to promote dendritic arborization. The interaction of PINK1 with VCP results in phosphorylation of the VCP co-factor NSFL1C/p47 at a novel site, eliciting more robust dendritic arbors. Mechanistically, PINK1 functions in a dual kinase/scaffolding role, activating PKA to phosphorylate p47. Given that mutations in PINK1 and VCP are known to cause Parkinson’s disease (PD) and frontotemporal dementia (FTD), conditions affecting primarily neurons, the discovery that they act in a common pathway to support dendritic arborization has important implications for neuronal health and disease.

## Introduction

Dendritic arborization and maintenance of appropriate connections are essential for neuronal health and function, while dendritic shrinkage is a feature of Alzheimer’s disease (AD), frontotemporal dementia (FTD) and Parkinson’s disease (PD; Patt et al., 1991; Terry et al., 1991; Ferrer, 1999; Lacor et al., 2007). Loss of function mutations in PTEN-induced kinase 1 (PINK1) are linked to recessive PD (Hatano et al., 2004; Valente et al., 2004a; Samaranch et al., 2010) and early-onset PD with dementia (PDD; 2007; Li et al., 2005; Ephraty et al., 2007; Ricciardi et al., 2014). Cortical atrophy and frontal-executive dysfunction are also observed in heterozygous carriers that have only partial loss of PINK1 (Reetz et al., 2008; Ricciardi et al., 2014), implicating PINK1 in an important neuroprotective role. Interestingly, PINK1 protects against a wide range of *in vitro* and *in vivo* insults (Valente et al., 2004b; Deng et al., 2005; Petit et al., 2005; Wang et al., 2007; Haque et al., 2008; Kamp et al., 2010), and neurons from *Pink1* knock-out mice exhibit dendritic shortening (Dagda et al., 2014).

PINK1 is a Ser/Thr kinase that is well known for its role in mitophagy (for review, see Cummins and Götz, 2018), although neurons can also use PINK1-independent mechanisms (Chu et al., 2013; Lin et al., 2017; McWilliams et al., 2018). PINK1 localizes with mitochondria (Silvestri et al., 2005; Gandhi et al., 2006; Dagda et al., 2009), but is also retrotransported to the cytosol (Beilina et al., 2005; Weihofen et al., 2008; Dagda et al., 2009) after processing by mitochondrial peptidases (Deas et al., 2011; Greene et al., 2012) for signaling or clearance. Basal levels of PINK1 vary by cell type and several factors regulate its stability and activity in the cytosol (Moriwaki et al., 2008; Weihofen et al., 2008; Murata et al., 2011; Matenia et al., 2012; Wang et al., 2014b; Aerts et al., 2015). While PINK1 has been extensively studied for its mitochondrial functions, recruiting Parkin to activate mitophagy (Matsuda et al., 2010; Narendra et al., 2010; Vives-Bauza et al., 2010) or regulating Complex I activity and mitochondrial calcium extrusion (Pridgeon et al., 2007; Morais et al., 2014; Kostic et al., 2015), less is known concerning its cytosolic functions. Cytosolic PINK1 is neuroprotective (Haque et al., 2008; Dagda et al., 2014), acting to regulate mTORC2 and Akt signaling (Murata et al., 2011).

The goal of the present study is to identify molecular pathways underlying the ability of PINK1 to promote dendritic arborization. Using an unbiased approach, we identified valosin-containing protein (VCP) as a major PINK1-binding protein. VCP is a multifunctional protein implicated in protein degradation, vesicular transport and dendritic spinogenesis (Forman et al., 2006; Ju et al., 2009; Wang et al., 2011; Kim et al., 2013; Shih and Hsueh, 2016). The diverse functions of VCP are determined by its various cofactors. The most studied VCP cofactors are the ubiquitin recognition factor in ER associated degradation 1 (UFD1)-nuclear protein localization homolog 4 (NPL4) dimer and p47 [also known as NSFL1 (p97) cofactor; NSFL1C]. The VCP-UFD1-NPL4 complex regulates protein degradation (Ye et al., 2001; Meyer et al., 2012), while the VCP-p47 complex controls membrane fusion and ER formation (Kondo et al., 1997; Shih and Hsueh, 2016). In the current study, we found that VCP and its cofactor p47 function downstream of PINK1 to promote dendritic outgrowth. Moreover, PINK1 functions as a PKA kinase, activating PKA to phosphorylate p47 at S176 to regulate dendritic complexity. Neither VCP nor p47 directly interact with the PKA holoenzyme, but PINK1 pulls down all components. These data support a novel molecular mechanism with the PINK1-VCP interaction acting to scaffold PKA-mediated phosphorylation of p47 and dendritogenesis.

## Materials and Methods

### Reagents

miVCP-GFP, sh-p47-GFP, GFP-VCP, MYC-VCP and VCP domains, MYC-p47, MYC-UFD1, PINK1-Flag, PINK1-GFP, GFP, ΔN-PINK1-GFP, OMM-GFP, OMM-PINK1-GFP, PKA_cat_-GFP, and AKAP-GFP were previously described (Dagda et al., 2014; Shih and Hsueh, 2016). MYC-p47(S176A) and MYC-p47(S176D) were constructed by site-directed mutagenesis. GST-TcPINK1 or GST-Parkin(UBL) were gifts of JF Trempe, McGill University, Canada. Antibody sources: FLAG (RRID:AB_259529); GAPDH (RRID:AB_732652), PKA_Cat_(pT197) (RRID: AB_2300165), PKA_reg_ (RRID: AB_777289); VCP (RRID: AB_2214638), GFP (RRID: AB_221570), PKA_reg_(pS96) (Thermo Fisher; RRID: AB_310220); Ki67 (RRID: AB_1140752), PKA_cat_ (RRID: AB_2170328); p47 (Novus, catalog #NBP2-13677), UFD1 (RRID: AB_11056444), PINK1 (RRID: AB_10127658).

### Cell culture

Cortical neurons from embryonic day E16 C57BL/6 mouse or E18 Sprague Dawley rat embryos were dissected and cultured in Neurobasal media containing 2% B_27_, and 2 mM Glutamax as previously described (Van Laar et al., 2011; Dagda et al., 2014) using procedures approved by the University of Pittsburgh Institutional Animal Care and Use Committee (IACUC). For some experiments, E16 cortical neurons were derived from *Pink1-/-* mice (Dagda et al., 2011, 2014; Zhi et al., 2018). Human HEK293 (ATCC catalog #CRL-1573, RRID:CVCL_0045); human neuronal SH-SY5Y [parental (ATCC catalog #CRL-2266, RRID:CVCL_0019), stable PINK1-3xFLAG (#24) and stable empty vector (M14) lines (Dagda et al., 2009)]; and the mouse neuronal HT22 line used for validation of mouse RNAi (RRID:CVCL_0321), were cultured and transfected using Lipofectamine 2000 as described (Dagda et al., 2009).

### Immunoprecipitation (IP), immunoblot, MS analysis

Lysates from HEK293 cells transfected with EGFPc-1 or PINK1-GFP were IPed using μMACS Epitope Tag Protein Isolation kit (Miltenyi Biotec). Endogenous proteins were IPed from HEK cells or from postmortem human cortex using 50 μl of Protein A and Protein G agarose beads (Invitrogen), 2 μg of antibody, and 1 mg of cell lysate overnight at 4°C. Beads were pelleted at 4500 × *g*, washed with TPBS, eluted using μMACS elution buffer, and resolved from Ig bands by electrophoresis through non-reduced gels for PINK1 and reduced gels for VCP. 2-D gels (IPG strips pH 3–11) were transferred to 0.45 μm PVDF for immunoblot analysis using the LI-COR Odyssey Fc imager as previously described (Cherra et al., 2010; Wang et al., 2014a).

For LC-MS, gel bands were excised, destained in 100 mM ammonium bicarbonate, reduced in 5 mM DTT, and alkylated in 15 mM iodoacetamide as previously described (Bateman et al., 2010). Gel spots were washed in 50% acetonitrile/100 mM ammonium bicarbonate and incubated overnight in 0.05 μg/μl trypsin in 100 mM ammonium bicarbonate at a 1:100 enzyme to protein ratio. Digested peptides were extracted in 60% acetonitrile/0.1% trifluoroacetic acid before being lyophilized and resuspended in 5% formic acid. Five replicate injections of sample digests were resolved by nanoflow reverse-phase liquid chromatography (EASY-nLC II, Thermo Fisher) coupled online via electrospray ionization to an Orbitrap Elite Mass Spectrometer (Thermo Fisher) configured to collect high-resolution peptide precursor measurements (*R* = 60,000 at 400 m/z) and to dynamically select the top 10 molecular ions for tandem MS via collision induced dissociation. Resulting data files were searched using SEQUEST (version 2.7) against a Uniprot-derived human proteome database (downloaded 01/17/2011) amended with an entry for green fluorescent protein (GFP) using the following settings: tryptic enzyme constraints (KR), two missed cleavage sites, peptide precursor mass tolerance of 10 ppm, fragment ion mass tolerance of 0.36 Da, variable modifications for methionine oxidation (15.99492 m/z), and static modifications for carboxyamidomethylation of cysteine (57.02146 m/z). Peptide false discovery rate (FDR) was determined with Percolator (version 2.1) using data file search results obtained from a decoy/scrambled reference database search (Käll et al., 2007). Peptide identifications exhibiting a *q* value ≤ 0.01 were considered confident identifications and prioritized for further analyses. Fold-change (Log2) ratios were calculated following addition of 0.5 to summed peptide spectral matches (PSMs) for corresponding protein identifications to enable comparative analyses of “presence-absence” scenarios (Käll et al., 2007; Eidhammer et al., 2012). The Orbitrap Fusion (capillary LC-MS/MS) instrument operated in positive ion mode was used for phosphopeptide identification. MSn was performed using ion trap mode to ensure the highest signal intensity of MSn spectra using both CID (for 2+ and 3+ charges) and ETD (for 4+–6+ charges) methods. The AGC Target ion number for ion trap MSn scan was set at 1000 ions, maximum ion injection time was set at 100 ms, and micro scan number was set at 1. The CID fragmentation energy was set to 35%. Sequence information from the MS/MS data were processed by converting the raw files into a merged file (.mgf) using RAW2MZXML_n_MGF_batch (merge.pl, a Perl script). The resulting files were searched using Mascot Daemon by Matrix Science version 2.3.2 against the most recent SwissProt or NCBI databases. A decoy database was also searched to determine the FDR, and peptides were filtered according to the FDR. The significance threshold was set at *p* < 0.05. Phosphorylated peptides were manually validated.

### Neuronal image analysis

Cells were fixed in 4% paraformaldehyde at room temperature (RT) for 15 min, washed twice with DPBS, and permeabilized in PBS containing 0.1% Triton X-100, for 30 min, then blocked in SuperBlock (Thermo Scientific) for 1 h. Primary antibodies were applied overnight at 4°C. Cells were washed 3× and incubated for 1 h with Alexa Fluor 488- or Alexa Fluor 546-conjugated secondary antibodies (Invitrogen) and PBS washed 3× before imaging on an Olympus IX71 inverted microscope, objective lenses of 40× and 60× magnifications (NA 1.3), using a DP80 camera and Olympus cellSens V1.17 software. Neurite length of undifferentiated SH-SY5Y cells was quantified as previously described (Chu et al., 2009). ImageJ was used to create binary skeletons (NeuronJ plugin) for Sholl Analysis (ShollAnalysis plugin), which utilizes consecutive-circle intersection analysis to quantify ramification richness and dendritic branching patterns (Sholl, 1953; Ristanovic et al., 2006). Briefly, following construction of concentric and equidistantly organized circles centered around the soma, the numbers of intersections of dendrites with circles of increasing radii were determined by computer assisted counting with manual verification. Data were analyzed by area under the curve (AUC; Pallotto et al., 2012) and linear Sholl analysis (number of dendritic intersections vs the circle radius; Benavides-Piccione et al., 2006).

### Computational modeling

Homology models for human PINK1 (hPINK1) were generated using Robetta based on the resolved structure of phPINK1 (PDB: 6EQI; Kim et al., 2004). Protein-protein docking simulations were performed using ClusPro (Kozakov et al., 2017). The top four hPINK1 models in the phosphorylated (S228p and S402p) forms were used as receptors (Aerts et al., 2015); the structure of PKA_cat_ in the Type IIa holoenzyme crystal (PDB ID: 2QVS) was input as ligand for docking simulations. For each docking simulation, up to 30 hPINK1-PKA_cat_ models were generated by ClusPro and rank-ordered by docking score (Kozakov et al., 2017). The complex structure with the largest docking occupancy (top lowest binding affinity) was selected. ATP and two Mg^2+^ ions, adopted from a phosphorylase kinase (PDB:2PHK) were docked to hPINK1 in the complex through structure alignment. The generated structure was further refined in solution (0.15 M NaCl) using molecular dynamics (MD) simulations of 100 ns. The root-mean-square deviation of C^α^-atoms from the original structure converged to 4.0 ± 0.5 Å after 30 ns, indicating the stability of the system.

### *In vitr*o kinase assay

GST-huPINK1, GST-TcPINK1, or GST-Parkin (UBL) expressed in BL21 *Escherichia coli* cells were induced by IPTG and purified with glutathione-agarose beads. *In vitro* phosphorylation reaction was performed with 1–2 μg purified recombinant protein, the indicated kinase, 200 μM ATP and 300 μCi/μmol γ-32P-ATP (PerkinElmer) in 1× protein kinase NE buffer (New England BioLabs, 50 mM Tris-HCl, 10 mM MgCl_2_, 0.1 mM EDTA, 2 mM DTT, and 0.01% Brij 35; pH 7.5) with phosphatase inhibitors (NaPi + Na_3_VO_4_) and incubated at 300°C for 1–2 h. Reactions were terminated by adding 10 μl 4× SDS-PAGE sample buffer and heat denaturing samples at 75°C for 20 min. Following 4–15% SDS-PAGE, protein bands were visualized with Coomassie Blue R-250 staining, then the gel was dried, and ^32^P incorporation was measured by autoradiography.

### Statistical analyses

Sholl data were analyzed using repeated measures ANOVA followed by *post hoc* Bonferroni-corrected AUC comparisons. The statistical package in GraphPad Prism was used for two-way and repeated measures ANOVA. The Bonferroni correction was applied for *post hoc* multiple comparison testing. Unpaired Student’s *t* test was performed using Microsoft Excel 2010 Analysis ToolPak.

**Figure 1.**
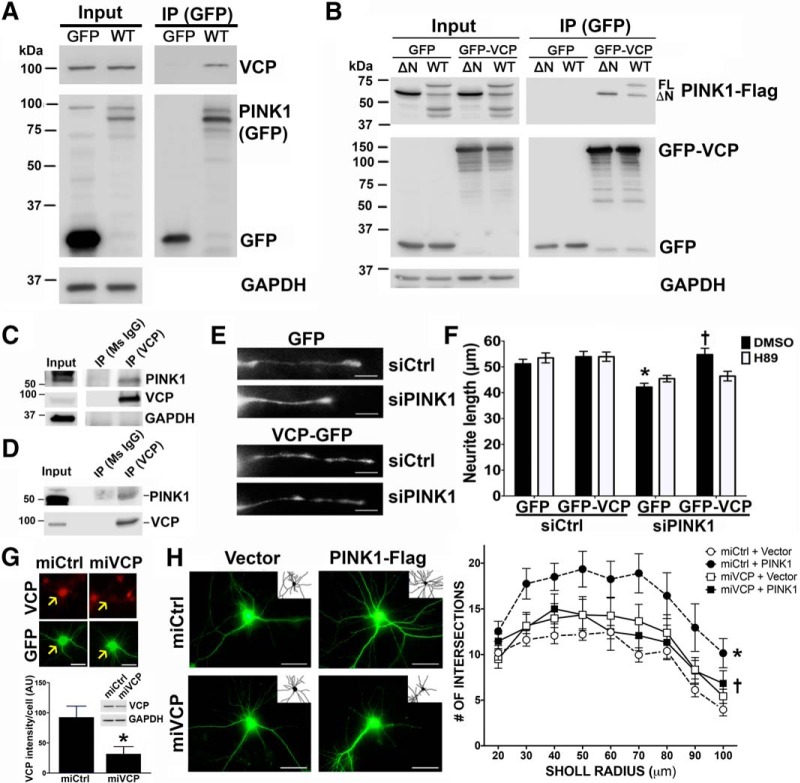
VCP interacts with PINK1 and is required for PINK1-mediated dendritogenesis. ***A***, Immunoblot of GFP IP products of HEK293 cells expressing GFP or WT-PINK1-GFP show that PINK1-GFP pulls down endogenous VCP (representative of five independent experiments). ***B***, GFP IP products of HEK293 cells co-expressing GFP or GFP-VCP and ΔN-PINK1-FLAG or WT-PINK1-FLAG show that GFP-VCP pulls down both full-length and processed PINK1 (representative of three independent experiments). ***C***, IP of endogenous VCP probed for endogenous PINK1 from HEK293 cells. ***D***, IP of endogenous VCP probed for endogenous PINK1 from human brain cortex. ***E***, ***F***, SH-SY5Y cells were co-transfected with scrambled control (siCtrl) or PINK1 siRNA (siPINK1) and with either GFP or GFP-VCP. Representative images of neurites are shown (***E***, scale bars = 10 μm), oriented with the somatic-neuritic junction to the left. ***F***, Quantification reveals that GFP-VCP rescues neurite shortening induced by siPINK1 (mean ± SEM; four independent experiments, 196–222 cells/group; *p < 0.0001, siPINK1 + GFP vs siCtrl + GFP; †*p* < 0.0001, siPINK1 + GFP-VCP vs siPINK1 + GFP. In SH-SY5Y cells, siPINK1 reduced expression of PINK1 mRNA to 49 ± 2.5% of siCtrl by Q-RT-PCR (*N* = 4). ***G***, VCP knock-down in mouse primary cortical neurons was verified by immunofluorescence for VCP (red). Arrows indicate transfected neurons (green; scale bars = 25 μm). Fluorescence intensity for VCP was quantified in neurons seven days after transfection with miCtrl versus miVCP (*p = 0.0006, representative of two independent experiments). Protein and mRNA knock-down were also confirmed in mouse neuronal HT22 cells 24 h after transfection, with mRNA reduced to 41.6 ± 2.2% of control cells by Q-RT-PCR (three independent experiments). Western blotting (inset) and band densitometry reveals that miVCP reduced VCP protein expression to 43.9–52.3% of miCtrl in two independent experiments. ***H***, Representative images and dendrite tracings (insets) of cortical neurons (scale bars = 50 μm) transfected with Vector or PINK1-Flag and miCtrl or miVCP. Sholl analyses reveals that the ability of PINK1 to increase dendritic complexity is inhibited by knock-down of VCP (mean ± SEM; 10–13 neurons/group, three independent experiments; *R* ^2^ = 0.2138, *F*_(3,44)_ = 3.9899; *p = 0.0015, miCtrl + PINK1 vs miCtrl + vector; †*p* = 0.0189, miVCP + PINK1 vs miCtrl + PINK1).

## Results

### VCP interacts with PINK1 and is required for PINK1-mediated dendritogenesis

To define potential mechanisms by which PINK1 regulates dendritogenesis, an unbiased label-free shotgun proteomic approach was employed to identify proteins with which PINK1 interacts. Lysates from HEK293 cells expressing GFP or PINK1-GFP were subjected to GFP-IP and the IP products analyzed by LC-MS. As expected, PINK1 shows the highest log enrichment in PINK1-GFP compared to GFP IP samples (Table 1), while GFP was identified at equivalent levels. VCP was among the two most highly enriched proteins co-IPed by PINK1-GFP (sequence coverage 24.194%), with a Log_2_ ratio comparable to that of CDC37, a component of the HSP90A chaperone system that is known to bind PINK1 and regulate its stability (Moriwaki et al., 2008; Weihofen et al., 2008). Expected PINK1 interactions with the mitochondrial import machinery (Bertolin et al., 2013) and proteasomal PSM subunits (Dächsel et al., 2005) were also confirmed, supporting the validity of the screen.

**Table 1:** PINK1-interacting proteins identified by IP-MS analysis

Gene name	Description	GFP-IP PSM	PINK1-GFP -IP PSM	Log2 ratio (PINK1-GFP/GFP)
PINK1	PTEN-induced kinase 1	0	415	9.70
CDC37	Cell division cycle 37 homolog	0	47	6.60
VCP	Valosin-containing protein	0	44	6.49
PSMB5	Proteasome subunit, beta type, 5	0	39	6.30
TIMM50	Translocase of inner mitochondrial membrane 50 homolog	0	22	5.49
TOMM40	Translocase of outer mitochondrial membrane 40 homolog	0	19	5.30
GFP	Green fluorescent protein	224	204	–0.13

PINK1-interacting proteins identified by a label-free mass spectrometry screen of GFP and PINK1-GFP IP products. The data were SEQUEST searched against the Uniprot human proteome database supplemented with the GFP sequence (P42212). Log2 ratios were calculated as described in Materials and Methods from the total peptide-spectral match counts in PINK1-GFP versus GFP control reactions. Top ranked candidates and known PINK1 interacting proteins are shown, all of which exhibited ratios >2 SD from the mean ratio of the entire dataset.

To confirm that PINK1 interacts with VCP, PINK1-GFP was IPed from HEK293 lysates and immunoblotted for endogenous VCP (Fig. 1*A*). In reciprocal IPs, GFP-VCP pulled down both full-length (FL) and processed (ΔN) forms of PINK1-FLAG (Fig. 1*B*). Furthermore, endogenous VCP interacts with endogenous PINK1 in both HEK293 cells (Fig. 1*C*) and in the human frontal cortex (Fig. 1*D*). Knock-down of PINK1 results in neurite shortening in SH-SY5Y cells, which was reversed by VCP overexpression (Fig. 1*E*,*F*). The rescue effects of VCP on neurite length were not due to changes in SH-SY5Y proliferation (Table 2). These data confirm a functional as well as physical interaction of PINK1 and VCP.

**Table 2:** VCP overexpression does not alter the proliferative fraction of SH-SY5Y cells

siRNA	GFP (mean ± SD)	VCP-GFP (mean ± SD)	*p* value
siCtrl	0.62 ± 0.02	0.59 ± 0.03	0.16
siPINK1	0.47 ± 0.03	0.46 ± 0.04	0.63

SH-SY5Y cells were co-transfected under the same conditions as in Fig. 1*E*. The cells were fixed after 40 h and stained for the proliferation antigen Ki-67. The proliferative fraction was calculated as the number of Ki-67 immunoreactive transfected cells divided by the total number of transfected cells (Student’s *t*-test, *n*=5). Transfection with VCP did not significantly affect cell cycle in either siCtrl or siPINK1 cells.

As we previously found that PKA regulates neurite morphology downstream of PINK1 (Dagda et al., 2014), we studied whether or not PKA could also be involved in VCP-mediated neurite protection. The PKA inhibitor H89 blocked the ability of VCP to rescue neurite shortening in PINK1-deficient cells (Fig. 1*F*), implicating PKA in this pathway. To determine whether VCP is necessary for the ability of PINK1 to increase dendritic complexity, we used a miRNA construct targeting rodent VCP (Wang et al., 2011), which co-expresses emerald GFP to outline the morphology of transfected neurons (miVCP; Fig. 1*G*), also validating knock-down biochemically using the mouse HT22 cell line (Fig. 1*G*, inset). Expression of the miVCP plasmid did not cause significant cell death in transfected neurons, but it inhibited the increased dendritic arborization induced by overexpression of PINK1 (Fig. 1*H*). Taken together, these data indicate that VCP is a necessary component in the PINK1 pathway regulating dendritic complexity.

### Processed PINK1 interacts with the D1 domain of VCP to regulate neurite extension

**Figure 2.**
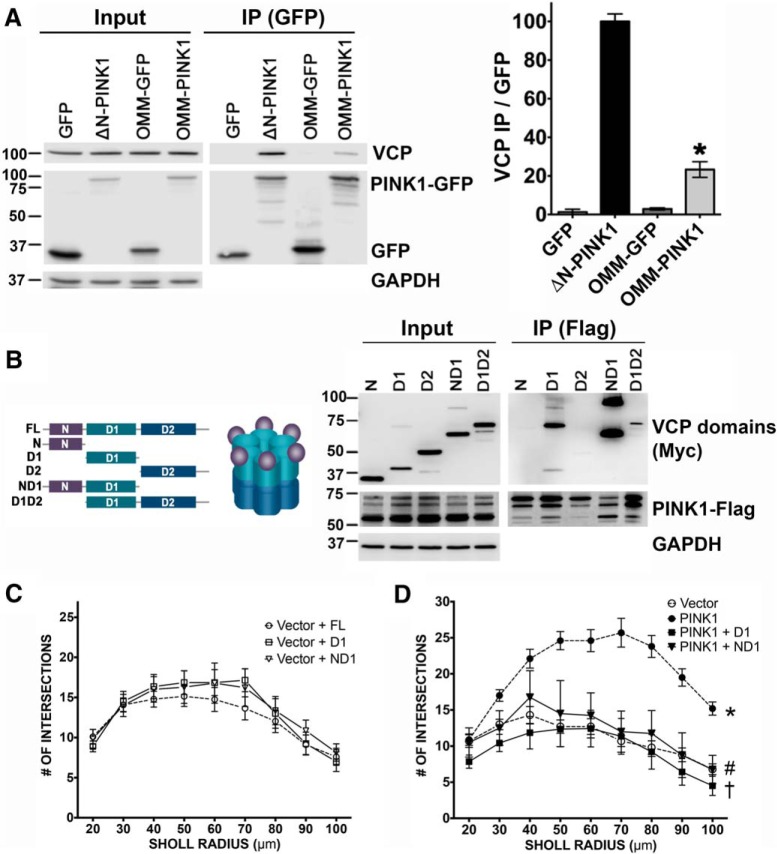
VCP favors cytoplasmic PINK1, binding via the D1 or ND1 domains of VCP. ***A***, GFP IP products from HEK293 cells expressing GFP, ΔN-PINK1-GFP, OMM-GFP, or OMM-PINK1-GFP. Co-IPed VCP was normalized to the density of IPed GFP, revealing that endogenous VCP is preferentially pulled down by ΔN-PINK1-GFP (mean ± SEM; three independent experiments, *p < 0.0001, OMM-PINK1 vs ΔN-PINK1). ***B***, Schematic of VCP truncation constructs. FLAG IP products from HEK293 cells expressing the MYC-tagged VCP domain constructs and PINK1-FLAG reveal that PINK1 pulls down D1, ND1, and D1D2 domains of VCP (representative of four independent experiments). ***C***, ***D***, Sholl analysis of dendritic arbors in primary neurons transfected as indicated reveal that overexpression of the isolated D1 or ND1 domain of VCP inhibits the ability of PINK1 to increase dendritic complexity (mean ± SEM; 10–14 neurons/group, three independent experiments; *R* ^2^ = 0.4706, *F*_(7,65)_ = 8.2530; *p* = 0.4475, vector+D1 vs vector+FL; *p* = 0.4700 vector+ND1 vs vector+FL; *p < 0.0001, PINK1 vs vector; †*p* < 0.0001, PINK1+D1 vs PINK1; #*p* = 0.0001, PINK1+ND1 vs PINK1).

Mitochondrially localized pools of PINK1 are required for many of its regulatory effects on mitochondrial homeostasis (Pridgeon et al., 2007; Matsuda et al., 2010; Narendra et al., 2010; Vives-Bauza et al., 2010; Zhang et al., 2013; Morais et al., 2014; Kostic et al., 2015), but not for all of its neuroprotective activities (Haque et al., 2008; Dagda et al., 2011; Murata et al., 2011). Likewise, VCP may be recruited to the outer membrane of depolarized mitochondria (Kim et al., 2013), although its most studied functions involve cytosolic interactions (Rabinovich et al., 2002). To determine whether mitochondrial localization is required for the interaction of PINK1 with VCP, PINK1 constructs lacking a mitochondrial targeting sequence (ΔN-PINK1-GFP) were compared to a previously described form of PINK1 engineered for constitutive expression at the outer mitochondrial membrane (OMM-PINK1-GFP; Dagda et al., 2014). Despite roughly equivalent levels of the two forms of PINK1 in both input and pulldown, ΔN-PINK1-GFP pulled down approximately four times more VCP than OMM-PINK1-GFP (Fig. 2*A*). These data suggest that mitochondrial localization is not required for PINK1-VCP binding, in accord with previous studies implicating cytosolic PINK1 in Akt signaling and cAMP-driven neuronal differentiation (Murata et al., 2011; Dagda et al., 2014).

To identify the region of VCP that binds to PINK1, a series of Myc-tagged VCP domain constructs were co-expressed in HEK293 cells and co-immunoprecipitated with PINK1-3xFLAG. The N-terminal domain was insufficient for VCP-PINK1 binding, but all of the constructs that contained the D1 ATPase domain were able to bind PINK1 (Fig. 2*B*). The D1 domain is known to mediate nucleotide-independent multimerization of VCP (Song et al., 2003). Interestingly, the ND1 fragment and the dimer form of D1 were pulled down by PINK1 to a greater degree than monomeric D1. In contrast, the isolated D2 domain did not interact with PINK1.

As the D2 domain of VCP is thought to be responsible for driving most functions associated with VCP (Song et al., 2003), we reasoned that the isolated, functionally inactive, ND1 and D1 domains could be used to interrupt PINK1 binding to endogenous VCP. Neither domain showed a basal effect on dendrite complexity in control vector-transfected neurons (Fig. 2*C*). However, co-expression of either D1 or ND1 inhibited the ability of PINK1 to promote dendritic complexity (Fig. 2*D*). These data indicate that the interaction of VCP with PINK1 is a necessary component of PINK1-induced neurite outgrowth.

### PINK1 and PKA regulate phosphorylation of the VCP co-factor p47

**Figure 3.**
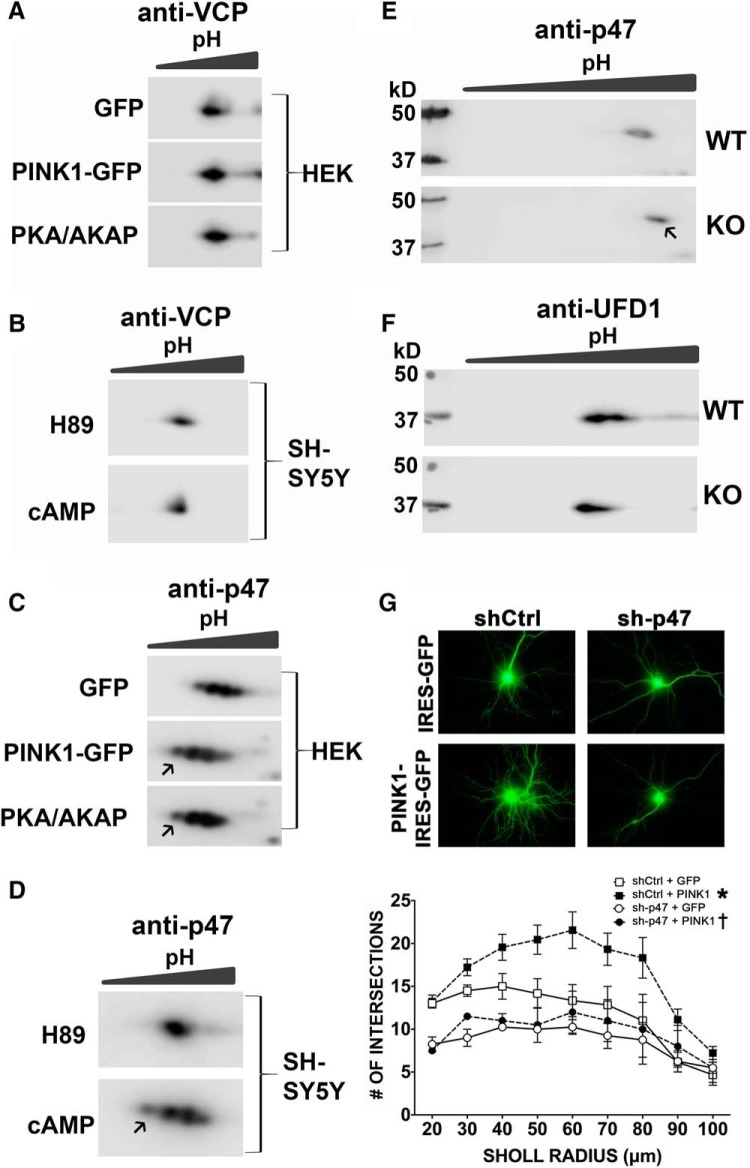
PINK1 and PKA alter isoelectric focusing of p47, a VCP adapter that is required for PINK1 induced dendritic outgrowth. HEK293 cells expressing GFP, PINK1-GFP, or co-expressing PKA_cat_-GFP and AKAP-GFP (PKA/AKAP) were analyzed by 2-D gel and blotted for VCP (***A***) and p47 (***C***). SH-SY5Y cells treated with 10 μg/ml H89 or 2.5 μM cAMP for 20 min were analyzed by 2-D gel and blotted for VCP (***B***) and p47 (***D***). While these treatments had no effect on VCP migration (***A***, ***B***), they elicited acidic shifts in p47 (***C***, ***D***, arrows) consistent with increased phosphorylation. ***E***, ***F***, Brain cortex from *Pink1* wild type (WT) or knock-out (KO) mice were analyzed by 2-D gel and blotted for p47 (***E***) or UFD1 (***F***). This reveals a basic shift (***E***, arrow) in p47 in *Pink1* knock-out brain tissues, consistent with decreased phosphorylation. Representative images and Sholl analysis of mouse neurons analyzed for the effects of shRNA knock-down of p47 (sh-p47; ***G***) reveal that knock-down of p47 inhibits the ability of PINK1 to promote dendritic arborization (*R* ^2^ = 0.6338, *F*_(3,18)_ = 10.3864; *p = 0.0029, shCtrl + PINK1 vs shCtrl + GFP; †*p* = 0.0016, sh-p47 + PINK1 vs shCtrl + PINK1).

To examine whether VCP may be a direct or indirect target of PINK1, we analyzed lysates from PINK1-GFP-transfected HEK cells compared to control GFP-transfected cells by 2-D-immunoblotting. There were no acidic shifts in isoelectric focusing to suggest phosphorylation of VCP (Fig. 3*A*). Although PKA is implicated in VCP-mediated neuroprotection (Fig. 1*F*), there was also no effect of PKA/AKAP overexpression on the VCP isoelectric point (Fig. 3*A*). Likewise, neither inhibiting nor stimulating PKA resulted in VCP isoelectric shifts in the neuronal SH-SY5Y cell line (Fig. 3*B*). In contrast, overexpression of either PINK1 or PKA in HEK cells elicited more acidic forms of the VCP co-factor p47 (Fig. 3*C*). Similarly, application of dibutyryl-cAMP to activate PKA in SH-SY5Y cells resulted in the appearance of more acidic forms of p47 (Fig. 3*D*). Using *Pink1* knock-out mice, we found that the loss of endogenous Pink1 resulted in basic shifts in mouse p47 (Fig. 3*E*), consistent with reduced phosphorylation. In contrast, there were no significant effects of Pink1 deficiency on the isoelectric point of another VCP co-factor, Ufd1 (Fig. 3*F*).

To determine whether or not p47 plays a role in PINK1-mediated increases in dendritic complexity, we performed Sholl analysis of primary mouse cortical neurons transfected with PINK1-GFP in the presence of shRNA targeting p47 (sh-p47). We found that the ability of PINK1 to promote dendritic arborization was reversed by sh-p47 (Fig. 3*G*), indicating that both VCP (Fig. 1*H*) and its co-factor p47 (Fig. 3*G*) are required for the ability of PINK1 to promote dendritic complexity.

**Figure 4.**
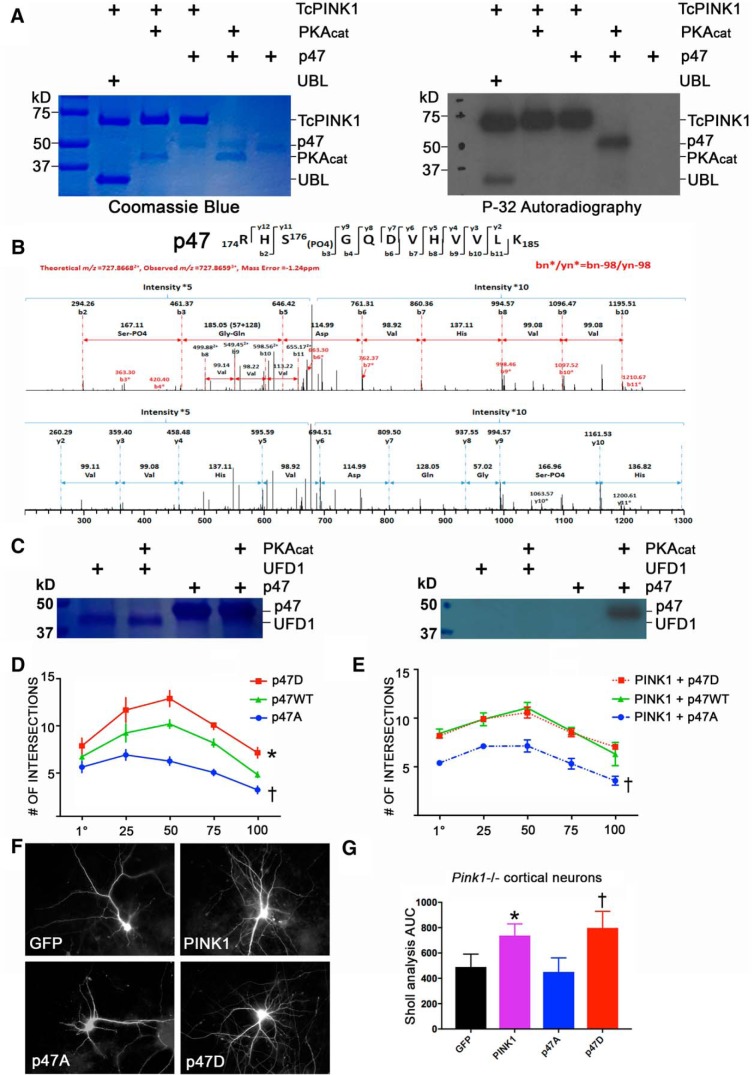
PKA phosphorylates p47 at a novel site that regulates dendritic complexity. ***A***, TcPINK1 and PKA_cat_ *in vitro* phosphorylation products visualized by Coomassie Blue staining and autoradiography reveal that PKA_cat_, but not TcPINK1 phosphorylates p47. ***B***, The p47 protein band from a non-radioactive kinase reaction was excised and analyzed by mass spectrometry, revealing a PKA-dependent p47 phosphopeptide at S176. ***C***, PKA_cat_ *in vitro* phosphorylation products visualized by Coomassie Blue staining and autoradiography reveal that p47, but not UFD1, is phosphorylated by PKA_cat_. ***D***, Sholl analysis of rat primary cortical neurons expressing the indicated forms of p47 indicate that p47D increases and p47A decreases dendritic complexity. (mean ± SEM; five independent experiments, *R* ^2^ = 0.7750, *F*_(2,12)_ = 20.6696, *p = 0.0139, p47D vs WT; †*p* = 0.0041, p47A vs WT). ***E***, Sholl analysis of rat primary cortical neurons co-expressing PINK1 and the indicated forms of p47 suggest that phosphorylation of p47 is necessary for the ability of PINK1 to increase dendritic complexity (mean ± SEM; five independent experiments, *R* ^2^ = 0.8842, *F*_(2,8)_ = 30.5674, *p* = 0.8544, PINK1+p47D vs PINK1+WT; †*p* = 0.0002, PINK1+p47A vs PINK1+WT). ***F***, ***G***, Representative images and Sholl analysis of primary cortical neurons from homozygous *Pink1-/-* mice transfected with GFP, PINK1, p47A, or p47D reveal that either PINK1 or p47D are able to rescue dendritic simplification in Pink1-deficient neurons (mean ± SEM; three independent experiments, *R* ^2^ = 0.8732, *F* = 20.6658, *p* = 0.6469, p47A vs GFP; *p = 0.0143, PINK1 vs GFP; †*p* = 0.0004, p47D vs GFP and *p* = 0.0069, p47D vs p47A).

To determine whether p47 may be a direct substrate of PINK1, we performed *in vitro* phosphorylation assays using the catalytically active *Tribolium castaneum* PINK1 (TcPINK1), which has previously been used to identify direct targets of PINK1 such as ubiquitin, Parkin and mitofilin (Matsuda et al., 2010; Kondapalli et al., 2012; Koyano et al., 2014; Tsai et al., 2018). Although TcPINK1 was active in phosphorylating the positive control UBL domain of Parkin, it did not phosphorylate p47 (Fig. 4*A*). In contrast, the catalytic subunit of PKA (PKA_cat_) directly phosphorylated p47 (Fig. 4*A*) at serine 176, as subsequently determined by mass spectrometry (Fig. 4*B*). In contrast, PKA_cat_ did not phosphorylate UFD1 (Fig. 4*C*).

To evaluate the impact of p47 phosphorylation (pS176) on neuronal differentiation, we engineered phosphomimic and non-phosphorylatable mutants, p47-S176D (p47D) and p47-S176A (p47A), respectively. We found that compared with wild type p47 (p47WT), expression of the p47D phosphomimic increased dendritic complexity in cortical neurons, whereas the non-phosphorylatable p47A elicited decreased complexity (Fig. 4*D*). When co-transfected with PINK1, the p47D plasmid did not further increase dendrite complexity compared to PINK1 co-transfected with p47WT (Fig. 4*E*). The lack of an additive effect is consistent with p47 phosphorylation functioning downstream of PINK1. In contrast, expression of p47A inhibited the ability of PINK1 to promote dendritic arborization, suggesting that phosphorylation of p47 is important in PINK1-elicited dendritogenesis. Finally, using primary cortical neurons from *Pink1* knock-out mice, we found that the p47D phosphomimic was able to rescue dendritic simplification to a comparable degree as adding back PINK1 (Fig. 4*F*,*G*). Taken together, these data indicate that PINK1 regulates dendritic morphogenesis by promoting PKA-dependent phosphorylation of p47.

### In addition to binding the VCP-p47 complex, PINK1 interacts with and activates PKA

**Figure 5.**
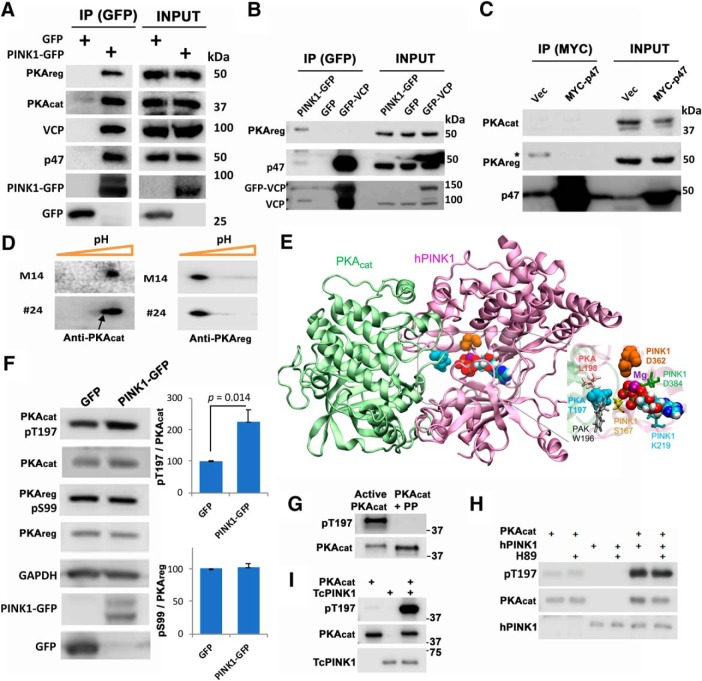
PINK1 phosphorylates the activation loop of PKA. ***A***, IP products of HEK293 cell lysates expressing GFP or PINK1-GFP reveal that PINK1 pulls down the PKA holoenzyme (PKA_cat_ and PKA_reg_) in addition to VCP and p47. ***B***, IP products of HEK293 lysates expressing PINK1-GFP, GFP or GFP-VCP reveal that either PINK1 or VCP pull down p47, but only PINK1 pulls down PKA_reg_. ***C***, IP products of HEK293 lystates expressing Vector or MYC-p47 reveals that p47 does not interact with PKA_cat_ or PKA_reg_. ***D***, 2-D analysis of endogenous PKA subunits in control (M14) and PINK1-3xFlag (#24) stable SH-SY5Y lines reveal an acidic shift of PKA_cat_ (arrow), but not PKA_reg_, in PINK1-overexpressing cells. ***E***, Structural model for the MD-equilibrated (100 ns) complex between hPINK1 (pink) and PKA_cat_ (green). PKA-T197 (cyan balls) and PINK1-D362 (orange balls) are shown in relation to PINK1-bound ATP with two Mg^2+^ (purple). Red, tan, white, cyan, and blue represent O, P, H, C, and N atoms, respectively, of ATP. ***F***, Immunoblot analysis of HEK293 cells transfected with GFP or PINK1-GFP reveals that PINK1-GFP increases PKA_cat_ phosphorylation at T197, but has no effect on PKA_reg_ phosphorylation at S99 (mean ± SD, three independent experiments, *p* = 0.014). ***G***, Recombinant PKA_cat_ purified from *E. coli*, which is already phosphorylated at T197, was dephosphorylated by incubation with λ phosphatase (PP) at 30°C × 1 h. Successful dephosphorylation was verified by immunoblot. ***H***, *In vitro* kinase assay of human GST-PINK1 with dephosphorylated recombinant PKA_cat_ in the presence or absence of the PKA inhibitor H89 reveals that hPINK1 phosphorylates PKA at T197. ***I***, *In vitro* kinase assay of GST-TcPINK1 with dephosphorylated recombinant PKA_cat_ reveals that TcPINK1 is also capable of directly phosphorylating PKA_cat_.

We have previously shown that PINK1 upregulates PKA activity within cells (Dagda et al., 2011, 2014). Several PINK1 modulated phosphoproteins are also direct targets of PKA (Sandebring et al., 2009; Kostic et al., 2015). To gain insight into the mechanism by which PINK1 promotes PKA activation, we studied the ability of PINK1 to interact with PKA in HEK cells. In addition to VCP and p47, we found that PINK1 could pull down both regulatory (PKA_reg_) and catalytic (PKA_cat_) subunits of PKA (Fig. 5*A*). In contrast, neither GFP, GFP-VCP or MYC-p47 were able to co-immunoprecipitate PKA (Fig. 5*B*,*C*).

As PINK1 modulates the isoelectric point of PKA_cat_, but not PKA_reg_ (Fig. 5*D*), we constructed a homology model for ATP-bound hPINK1 and performed docking simulations against the crystal structure of PKA_cat_. Interestingly, the PKA_cat_ activation loop was observed to make interfacial contacts with the PINK1 ATP-binding site, and MD simulations to verify the stability of the binding pose further showed that T197 in the PKA_cat_ activation loop underwent a rotational isomerization to expose its hydroxyl group toward the ATP binding site of PINK1 (Fig. 5*E*). As activation of PKA_cat_ is known to occur on phosphorylation of either PKA_cat_(T197) or PKA_reg_(S99) (Shoji et al., 1983; Manni et al., 2008; Taylor et al., 2013), we analyzed the phosphorylation status of these residues in cells transfected with PINK1. We found that PKA_cat_(pT197) levels were significantly increased in PINK1-GFP expressing cells, with no change in phosphorylation of PKA_reg_(S99) (Fig. 5*F*).

**Figure 6.**
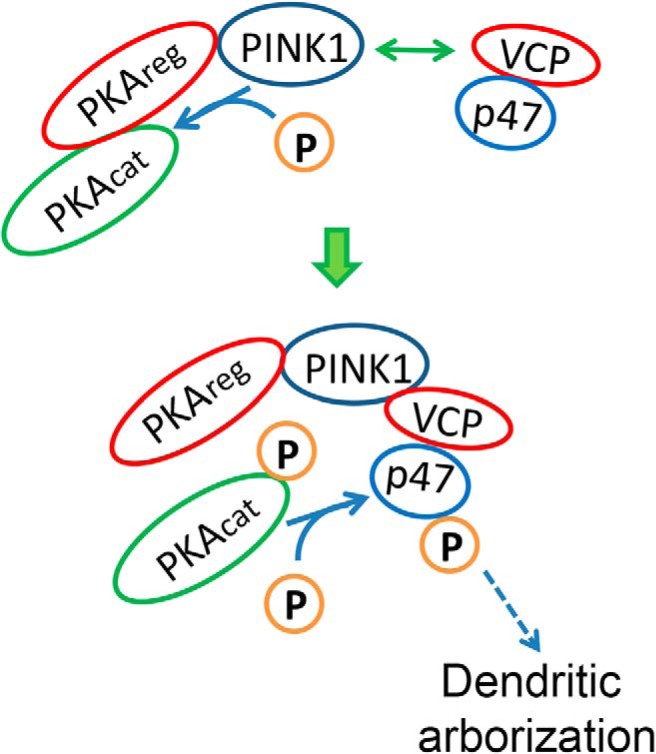
Schematic of PINK1- and VCP-dependent dendritic arborization. Working model for a dual kinase/scaffolding role for PINK1 in promoting p47 phosphorylation and increased dendritic complexity. PINK1 binds to the PKA holoenzyme and to the VCP-p47 complex, phosphorylating and activating PKA_cat_ in the vicinity of p47. This results in phosphorylation of p47 by PKA at a novel site, promoting dendritic growth and arborization.

To determine whether or not PINK1 is able to directly phosphorylate PKA_cat_ at T197, we performed *in vitro* kinase assays using recombinant PKA_cat_. As recombinant PKA_cat_ isolated from *E. coli* is known to be fully phosphorylated on T197 and S338 (Knighton et al., 1991), we treated recombinant PKA_cat_ with a phosphatase to produce a dephosphorylated substrate (Fig. 5*G*). Dephosphorylated PKA_cat_ was then incubated with a purified human GST-PINK1 recombinant protein for *in vitro* kinase reaction. Incubation with PINK1 significantly increased levels of PKA_cat_(pT197), and this increase was not inhibited by inclusion of the PKA inhibitor H89 (Fig. 5*H*). These data confirm that hPINK1 is able to directly phosphorylate the catalytic subunit of PKA. Likewise, although TcPINK1 did not further phosphorylate recombinant PKA_cat_ from *E. coli*, which is already fully phosphorylated (Fig. 4*A*; Knighton et al., 1991), it was able to phosphorylate the dephosphorylated PKA_cat_ at T197 (Fig. 5*I*). Therefore, we conclude that PINK1 can function as a novel kinase for PKA activation. We propose that PINK1 acts to bring together PKA and the VCP-p47 complex, facilitating PKA-mediated phosphorylation of p47 to promote neurite outgrowth and dendritic arborization (Fig. 6).

## Discussion

While PINK1 has been studied extensively for its role in mitochondrial quality control in a variety of cell types (Matsuda et al., 2010; Narendra et al., 2010; Vives-Bauza et al., 2010), mechanisms by which loss-of-function mutations in PINK1 result in neurodegeneration remain unclear. We have previously shown that loss of PINK1 results in dendritic simplification (Dagda et al., 2014). Here, we report that PINK1 interacts with VCP, p47 and PKA to promote dendritic arborization in neurons. Whereas VCP is essential for the ability of PINK1 to regulate dendritic complexity, there was no isoelectric shift to suggest VCP phosphorylation (Figs. 1–3). Instead, we found that PINK1 functions as a novel PKA kinase (Fig. 5), activating PKA to phosphorylate p47 (Fig. 4*A–C*). Interestingly, p47 regulates non-degradative aspects of VCP function such as protein synthesis and spinogenesis (Shih and Hsueh, 2016). Overexpression of the p47D phosphomimic was sufficient to mimic the effects of PINK1 overexpression (Fig. 4*D*), and to rescue dendrite complexity in *Pink1*-/- mouse neurons (Fig. 4*F*,*G*), whereas the nonphosphorylatable p47A blocked effects of PINK1 overexpression (Fig. 4*E*). These findings represent a novel function of PINK1 that may be particularly important for maintaining neuronal health and function.

The ability of PINK1 to regulate dendritic arborization in cells with functioning mitochondria is distinct from its activity in promoting autophagic clearance of depolarized mitochondria. Whereas localization of PINK1 at the outer mitochondrial membrane (OMM) is necessary and sufficient to trigger depolarization-triggered mitophagy (Kawajiri et al., 2010; Matsuda et al., 2010), OMM-targeted PINK1 fails to support dendritic outgrowth, acting instead to reduce mitochondrial density in dendrites (Dagda et al., 2014). This is not unexpected given the known role of autophagy in mediating neurite retraction (Yang et al., 2007; Plowey et al., 2008; Chu et al., 2009). In contrast, cytosolically localized ΔN-PINK1 promotes neurite outgrowth in SH-SY5Y cells and rescues dendritic simplification in *Pink1*-/- neurons (Dagda et al., 2014). Recently, VCP and its UFD1 cofactor have been implicated in mitochondrial quality control, translocating to mitochondria to regulate outer membrane protein proteasomal degradation and PINK1-regulated mitophagy (Kim et al., 2013; Guo et al., 2016; Guo and Qi, 2017; McLelland et al., 2018). In our neuronal system, there was no evidence of VCP mitochondrial localization. Our data also demonstrate involvement of p47, rather than the UFD1 cofactor, in regulating dendritic morphogenesis. Moreover, endogenous VCP was preferentially immunoprecipitated by ΔN-PINK (Fig. 2*A*), consistent with a predominant interaction in the cytosolic compartment. Several other cytosolic functions of PINK1 have been reported, including neuroprotection and the activation of Akt and PKA signaling pathways predicted to suppress autophagy (Haque et al., 2008; Murata et al., 2011; Dagda et al., 2014; Fedorowicz et al., 2014). Taken together, these data implicate mitochondrial health as a key metabolic switch regulating the subcellular location and pro-growth versus degradative functions of PINK1.

It has been established that, in contrast to *E. coli*, PKA activation in mammalian systems requires the action of a heterologous kinase with properties similar to phosphoinositide-dependent kinase-1 (Moore et al., 2002). Our findings that PINK1 is able to function as a PKA kinase adds mechanistic insight to the previously reported ability of PINK1 to upregulate phosphorylation of other PKA targets in cells, such as Drp1 and NCLX (Sandebring et al., 2009; Kostic et al., 2015). Interestingly, PINK1 pulls down PKA, VCP and p47, whereas neither VCP nor p47 could pull down PKA. We propose that PINK1 functions to bring PKA and VCP-p47 together, activating PKA and enabling phosphorylation of p47 to promote dendritogenesis in neurons (Fig. 6).

There are several mechanisms by which VCP, a hexameric protein with multiple functions, could regulate dendritic morphology. These include effects on membrane fusion and vesicular trafficking (Bug and Meyer, 2012) as well as proteasomal degradation of regulatory proteins (Xu et al., 2011; Meyer et al., 2012; Christianson and Ye, 2014). Whereas many of its degradative functions are related to its UFD1 cofactor (Meyer et al., 2000), the p47 cofactor has been implicated in pro-growth functions of vesicular transport, protein synthesis, Golgi and ER biogenesis, and spinogenesis (Shih and Hsueh, 2016; Kondo et al., 1997). Given the role of Golgi outposts in determining dendritic branch points (Koleske, 2013), it would be interesting to study the effects of p47D on Golgi distribution. VCP is also able to regulate dendritic spine density through its interactions with neurofibromin (Wang et al., 2011), a protein whose adult expression is limited to neurons, myelinating glia and adrenal medulla cells (Daston and Ratner, 1992). Interestingly, neurofibromin regulates not only PKA (Brown et al., 2010), but also CRMP2 (Lin and Hsueh, 2008; Patrakitkomjorn et al., 2008), a protein necessary for proper dendritic bifurcation (Niisato et al., 2013). The current data indicates that phosphorylation of the VCP cofactor p47 plays an important role in regulating dendritic outgrowth and complexity downstream of PINK1.

Further studies will be necessary to define which aspects of dendritic morphogenesis are regulated by this pathway. Dendritic arborization proceeds through several steps, including initiation and elongation of dendritic outgrowth, development of secondary and higher order branchpoints, self-avoidance or tiling of dendritic fields and spinogenesis balanced against processes of synaptic pruning and branch retraction (Ledda and Paratcha, 2017). Whereas these processes are quite dynamic developmentally, the major dendritic branches are generally stable in the mature nervous system with remodeling concentrated distally (Trachtenberg et al., 2002). In our system, it appears that VCP plays a greater role during the active phase of PINK1-stimulated arborization than in basal maintenance. In neurodegenerative disease states, however, processes that serve to suppress retraction and elimination of dendritic branches and spines are differentially impaired (Koleske, 2013), and the potential role of the PINK1-VCP-PKA-p47 signaling cassette in these contexts remain to be determined. Given the prevalence of dendritic pathology in neurodegenerative diseases, further study of these processes may offer important insights regarding how PINK1 and VCP function to prevent neurodegeneration.

In summary, we have delineated a novel mechanism by which PINK1 and VCP interact to promote PKA-mediated p47 phosphorylation and dendritic arborization. These data implicate a novel set of specialized neuronal functions for PINK1 and VCP, bringing together two proteins linked to different neurodegenerative diseases. Mutations in VCP contribute to FTD, amyotrophic lateral sclerosis, inclusion body myositis and Paget’s disease (for review, see Nalbandian et al., 2011); mutations in PINK1 elicit motor and cognitive dysfunction characteristic of early-onset PD/PDD (Li et al., 2005; Ephraty et al., 2007; Ricciardi et al., 2014). While these mutations are individually rare, their involvement in a common pathway regulating dendritic morphology offers novel insights into mechanisms that may be harnessed to support the development of therapies focused on maintaining the health and function of neuronal arbors.

## Synthesis

Reviewing Editor: Leonidas Stefanis, Biomedical Research Foundation of the Academy of Athens Decisions are customarily a result of the Reviewing Editor and the peer reviewers coming together and discussing their recommendations until a consensus is reached. When revisions are invited, a fact-based synthesis statement explaining their decision and outlining what is needed to prepare a revision will be listed below. The following reviewer(s) agreed to reveal their identity: NONE. Note: If this manuscript was transferred from JNeurosci and a decision was made to accept the manuscript without peer review, a brief statement to this effect will instead be what is listed below.

Overall, this is a very interesting paper that demonstrates the involvement of cytosolic PINK1 in a pathway together with PKA, VCP and p47, possibly in a protein complex, leading to enhanced dendritic arborization. These findings are novel and well substantiated by presented data. Compared to the original publication in J Neurosci, there are new data reinforcing the relevance of the findings in an in vitro and in vivo setting.

There are some minor issues:

It is unclear what is shown in Figure 1E. This has to be described in some detail in the Figure legend and a meaningful image needs to be presented. In 1G, quantification and statistics from multiple WBs need to be presented; alternatively, a statement that the experiment was repeated at least twice with similar results, as well as a quantification of the band intensity from the presented image need to be shown.

The figure legends are not very explanatory, and need to be much more detailed and explicit as to what is being shown.

There are some typos. For example, “ Finally, using primay cortical neurons from Pink1 knockout mice”
